# Protease-Based Subunit Vaccine in Mice Boosts BCG Protection against *Mycobacterium tuberculosis*

**DOI:** 10.3390/vaccines10020306

**Published:** 2022-02-16

**Authors:** Ana Paula Junqueira-Kipnis, Carine de Castro Souza, Ana Carolina de Oliveira Carvalho, Fabio Muniz de Oliveira, Vinnycius Pereira Almeida, Alisson Rodrigues de Paula, Mara Rubia Celes, André Kipnis

**Affiliations:** Department of Biosciences and Technology, Institute of Tropical Pathology and Public Health, Federal University of Goiás, Goiânia 74605-050, Brazil; ana_kipnis@ufg.br (A.P.J.-K.); carine-castro@hotmail.com (C.d.C.S.); anna.carvalho201348@gmail.com (A.C.d.O.C.); fabiomuniziptsp@gmail.com (F.M.d.O.); vinny.biotec@gmail.com (V.P.A.); alisson_br@outlook.com (A.R.d.P.); mrubia_celes@ufg.br (M.R.C.)

**Keywords:** tuberculosis, subunit vaccine, PepN, PepA/Mtb32a, Msh1, recombinant BCG, adjuvant, Advax4

## Abstract

The significant number of people with latent and active tuberculosis infection requires further efforts to develop new vaccines or improve the Bacillus Calmette-Guérin (BCG), which is the only approved vaccine against this disease. In this study, we developed a recombinant fusion protein (PEPf) containing high-density immunodominant epitope sequences from Rv0125, Rv2467, and Rv2672 *Mycobacterium tuberculosis* (Mtb) proteases that proved immunogenic and used it to develop a recombinant BCG vaccine expressing the fusion protein. After challenging using Mtb, a specific immune response was recalled, resulting in a reduced lung bacterial load with similar protective capabilities to BCG. Thus BCG PEPf failed to increase the protection conferred by BCG. The PEPf was combined with Advax4 adjuvant and tested as a subunit vaccine using a prime-boost strategy. PEPf + Advax4 significantly improved protection after Mtb challenge, with a reduction in bacterial load in the lungs. Our results confirm that Mtb proteases can be used to develop vaccines against tuberculosis and that the use of the recombinant PEPf subunit protein following a prime-boost regimen is a promising strategy to improve BCG immunity.

## 1. Introduction

Tuberculosis (TB) is an infectious disease caused by *Mycobacterium tuberculosis* (Mtb) and is the leading cause of death by a single infectious agent worldwide. In 2020, an estimated 1.5 million individuals died of TB, including about 200,000 HIV-positive individuals. Additionally, the emergence of first-line (MDR-TB) and second-line (XDR-TB) antibiotic-resistant strains of Mtb highlights the urgency for new vaccines to protect against pulmonary TB in adults [1,2]. According to the World Health Organization (WHO), until the SARS-CoV-2 pandemic, tuberculosis was the prime disease causing death via a single infectious agent. Due to the pandemic, the diagnosis, treatment, and reporting of TB cases/deaths have been negatively impacted. The WHO forecasts that the impact will be even greater, with drastic reductions in TB case notifications in 2021 and 2022. In September 2015, the WHO set a goal for TB eradication known as the End TB Strategy, which aims at a global reduction of TB deaths and TB incidence of 90% and 80%, respectively, by 2030 [2].

Vaccination is one of the most effective ways to prevent and eradicate infectious diseases [3]. Bacillus Calmette-Guérin (BCG) vaccine has been used to prevent TB for 100 years and is the only accepted form of prophylaxis against the disease [4]. The attenuation of the BCG strain was caused by the irreversible deletion of the RD1 region responsible for encoding crucial antigens for *M. tuberculosis* virulence, such as ESAT-6 and CPF-10. BCG is the most administered vaccine in children and has a worldwide vaccination coverage of 85%, including 22 high-burden countries [4,5]. It protects children and adolescents against meningeal and pulmonary TB, the severe forms of the disease. Despite being a functional and safe vaccine for children, its effectiveness is variable in adults due to vaccine protection lasting approximately 10 to 12 years, exposure to environmental mycobacteria possibly affecting the immune system, and differences among BCG strains possibly causing a loss of immunogenic determinants in the BCG vaccine [1,4,6].

Bioinformatics and reverse vaccinology offer valuable tools for the identification of proteins and peptides with high potentials of inducing tailored immune responses in different animal models and humans [7]. The analysis of proteins via immunoinformatics provides a more rational and guided method of identifying the amino acid sequences containing the highest number of immunogenic epitopes within each protein. Moreover, for the sake of developing a vaccine that can be immunogenic in various populations, a high diversity of human leukocyte antigens (HLAs) should be considered in the analysis [8]. Physicochemical properties, such as stability and hydrophobicity, are also critical factors that affect the feasibility of the antigen [9]. As such, exploring these tools is a cost-efficient and quicker way to identify the most promising protein sequences; however, they must be evaluated in vivo to confirm their capacity to prevent and control infection.

Mtb produces several proteases that are involved in the mycobacteria’s physiology and virulence. As they favor infection in the host [10], they may represent promising developmental vaccine targets. Among the proteases produced by Mtb, specific proteins, such as Rv0125 (PepA), Rv2467 (PepN), and Rv2672 (Msh1), have been highlighted due to their immunological and physiological proprieties. PepA, a serine protease, was observed to stimulate the proliferation of peripheral blood mononuclear cells from tuberculin skin-test-positive patients (latent TB individuals), indicating that Mtb expresses PepA during infection and is immunogenic [11]. Due to these properties, PepA is one of the proteins included in the sequence of M72/AS01E, a TB vaccine tested in a clinical trial phase IIb. A follow-up 3-year study revealed that the vaccine’s efficacy for preventing pulmonary TB among latently infected individuals was 49.7%, with adverse events similar to those of the placebo control [12].

The PepN protease is an aminopeptidase N of Mtb. In previous studies, PepN proved nonessential for Mtb growth in vitro, whereas, in a non-pathogenic mycobacterium, it was deleterious for growth [13]. Furthermore, interactome analyses of PepN from Mtb and *M. smegmatis* revealed that these proteins interact with different types of mycobacterial proteins that could be involved in the bacilli’s virulence. Thus, this protease is present in pathogenic and non-pathogenic mycobacteria, with varying physiological functions depending on the bacteria [10].

Finally, Msh1 is a mycobacterial-secreted hydrolase 1 that exhibits both lipase and protease activities. As Msh1 augments the capacity of Mtb to survive in a lipid-rich intracellular environment, it is essential for Mtb proliferation inside foamy macrophages; however, this property was not observed in regular culture conditions in vitro. Furthermore, this protease is highly expressed in lung homogenates during chronic Mtb infection in murine models [14].

Given the demonstrated importance of proteases PepA, PepN, and Msh1 (besides their immunogenic capacity) for the survival and virulence of Mtb, this work investigates the immunogenicity of a recombinant fusion protein composed of immunodominant epitopes from these proteases, development of a recombinant BCG vaccine and their use in different vaccination strategies for the prevention of TB in a murine model.

## 2. Materials and Methods

### 2.1. Prediction of MHC (Major Histocompatibility Complex) Class I and Class II Binding Epitopes and Fusion Protein Design

The FASTA sequence of the proteases Rv0125, Rv2467, and Rv2672 from Mtb H37Rv were retrieved from NCBI (https://www.ncbi.nlm.nih.gov, (accessed on 11 February 2022) and used for the selection of CD8 and CD4 epitopes using the Immune Epitope Database (IEDB) HLA-I and -II Binding Predictions servers (http://tools.iedb.org/mhci/ (accessed on 11 February 2022) and http://tools.iedb.org/mhcii/ (accessed on 11 February 2022), respectively). For HLA-I, the IEDB 2.19 recommended method was selected, and the allele reference list that was used is listed in Supplementary Table S1. Epitopes with ANN and SMM IC50 ≤ 500 nM of 9 amino acids were considered binding epitopes [15,16]. For HLA-II, the IEDB recommended method was also applied, and epitopes with percentile rank ≤ 10 were considered candidate-binding epitopes [17,18,19]. Considering the IEDB HLA-I and II (http://tools.iedb.org/main/ (accessed on 11 February 2022) binding prediction result tables, we made use of Python programming language to create an algorithm, the Vaccine-Associated Density Epitope Research (VADER): https://replit.com/@vaccine/VADER/ (accessed on 11 February 2022). VADER screens linear sequences from a given protein with a specified size and gives as output a graph in which the user can identify the epitope density throughout the entire sequence. Additionally, it is possible to compare (in the same graph), for example, the epitope density of different tables, such as for HLA-I and HLA-II or the major histocompatibility complexes (MHCs) from different species. The full HLA-II reference set was chosen for this selection (Supplementary Table S1). Next, all overlapping sequences (with a length of 81 amino acids) of the 3 proteases were assessed, and the sequence with the highest epitope density from each protease was selected to construct the chimeric protein (the first 35 amino acids were excluded from the analysis to avoid the signal peptide). Between each protease-derived sequence, a GSGSGS spacer was inserted to avoid neoantigens. The resulting protein was assessed regarding its biochemical parameters with ProtParam (https://web.expasy.org/protparam/ (accessed on 11 February 2022).

For in silico allergenicity prediction, AllergenFP (http://ddg-pharmfac.net/AllergenFP/ (accessed on 11 February 2022) [20], Algpred (http://crdd.osdd.net/raghava/algpred/ (accessed on 11 February 2022) [21], and AllerTOP (http://www.ddg-pharmfac.net/AllerTOP/index.html (accessed on 14 February 2022) [22] was used. Toxicity was predicted using ToxinPred (http://crdd.osdd.net/raghava/toxinpred/protein.php (accessed on 11 February 2022), which assigns an SVM toxicity score for each protein epitope based on its properties (e.g., hydropathicity, hydrophilicity, charge, etc.) [23].

### 2.2. Secondary and Tertiary Structure Prediction of PEPf

The secondary and tertiary structures of PEPf were generated using RaptorX (http://raptorx.uchicago.edu/ (accessed on 11 February 2022) [24]. The generated PDB file was refined using Galaxy Refine (http://galaxy.seoklab.org/ (accessed on 11 February 20221) [25]. The server generated five refined models, which were verified regarding the ψ and ϕ angles of their amino acids using Ramachandran plots. The structure with the lowest number of outliers was selected for representation.

### 2.3. Bacterial Strains Used in This Study

*Escherichia coli* XL1-Blue and BL21 (DE3) pLysS were used for cloning and expression of recombinant PEPf protein, respectively. *E. coli* strains were cultured in Luria Bertini (LB) broth or agar at 37 °C. *Mycobacterium bovis* BCG Moreau was cultivated in 7H11 agar supplemented with 10% Oleic Albumin Dextrose Catalase (OADC) and sodium pyruvate, and Middlebrook 7H9 medium with 10% OADC, sodium pyruvate, and 0.05% Tween 80 at 37 °C. When required, antibiotics were used at the following concentrations for the selection of *E. coli* recombinants: kanamycin (20 µg/mL) and chloramphenicol (20 µg/mL).

### 2.4. Expression and Purification of Recombinant PEPf Protein

The coding DNA sequence of PEPf protein was synthesized into the pET28(a)+ at sites *Nde*I and *Hind*III resulting in the pET28_PEPf (Genone, https://www.genone.com.br/ (accessed on 11 February 2022)). The pET28_PEPf was inserted into *E. coli* BL21 (DE3) pLysS cells by transforming for PEPf protein expression. *E. coli* BL21 (DE3) pLysS carrying pET28_PEPf were grown in LB containing kanamycin (20 µg/mL) and chloramphenicol (20 µg/mL) until optical density (OD) of 550 nm reached 0.5. After that, the culture was induced with 0.6 mM of isopropyl-1-thio-β-D-galactopyranoside (IPTG) at 37 °C, 180 rpm for 4 h. The cells were then harvested by centrifugation at 4000× *g* for 20 min at 4 °C. The pellet was used for protein extraction using the commercial protein purification QIAexpress-Ni-NTA Fast Start Kit (Qiagen, Carlsbad, USA), according to the manufacturer’s instructions. Fractions collected during protein purification were analyzed on 12% SDS-PAGE. The concentration of purified PEPf protein was determined by using Bradford’s reagent with bovine serum albumin as the standard.

### 2.5. Animal Care and Use

The study was conducted on BALB/c female mice, aged 6–8 weeks, obtained from the Institute of Tropical Pathology and Public Health of the Federal University of Goiás (UFG). During the experimental procedures, the animals were kept in isolators with HEPA filters, with constant temperature (24 ± 1 °C) and humidity (50 ± 5%) and under controlled light conditions (12 h of light and 12 h of dark). The animals received water and feed ad libitum. The animals were handled in accordance with the guidelines of the National Council for Animal Control and Experimentation (CONCEA). The study was approved by the Ethics Committee on Animal Use (CEUA; nº 076/20) of the UFG. All animals were evaluated daily for any signs of stress, and none of the animals presented signs of disease throughout the experimental period. To minimize the distress, environmental enrichment materials were used in each cage. If, at any point, an animal presented signs of disease such as anorexia or dehydration, the animal was humanly euthanized. The experiments were repeated and showed reproducibility.

### 2.6. PEPf Immunogenicity Evaluation Strategy

Briefly, immediately prior to vaccination, 1 mg of Advax4 kindly provided by Dr. Nikolai Petrovsky of Flinders University (Adelaide, Australia) was combined with 20 µg of recombinant PEPf protein. BALB/c mice were divided into four groups of four animals each. Groups were vaccinated with PBST, BCG, PEPf + Advax4 (subunit vaccine) or with Advax4 alone. The animals were vaccinated twice with 100 µL intramuscularly, with a 15-day interval between immunizations with PEPf + Advax4 or Advax4 alone. The BCG vaccine was administered once subcutaneously using the 10^6^ CFU on day one of the PEPf + Advax4 immunization. Fifteen days after the last immunization, the animals were euthanized by cervical dislocation by a veterinarian to evaluate the immune response elicited against PEPf. This experiment was repeated twice. In one of the experiments, an additional control group consisting of vaccination with PEPf alone was included to evaluate if the protein could induce an immune response without adjuvant.

### 2.7. Construction of BCG-PEPf

In order to express the PEPf protein in a live attenuated vector vaccine, pVV16-PEPf was generated by subcloning the *Nde*I-*Hind*III fragment corresponding to the *pepf* gene of pET28a-PEPf into the corresponding sites of the pVV16 vector [26]. Competent *Mycobacterium bovis* BCG Moreau cells were prepared as previously described [27]. The BCG-PEPf vaccine was obtained after electroporation of the pVV16-PEPf vector in *M. bovis* BCG Moreau. As a control, transformants with empty plasmids were obtained. Recombinant mycobacteria were tested on 7H11 agar supplemented with kanamycin (50 µg/mL) and hygromycin (150 µg/mL). The presence of a DNA sequence encoding the PEPf protein in the recombinant cells was analyzed by colony PCR using specific primers. Colonies positive for the presence of the PEPf gene were selected and cultivated in 7H9 medium supplemented with antibiotics at 37 °C until reaching the logarithmic phase. The expression of the *pepf* gene was analyzed via RNA extraction, cDNA synthesis, and PCR, as previously described [28]. After confirmation of PEPf protein expression by the BCG-PEPf vaccine, vaccine aliquots were produced by growing vaccine in a single batch in Middlebrook 7H9 medium supplemented with kanamycin (50 µg/mL) and hygromycin (150 µg/mL) until reaching the growth of the log phase.

### 2.8. Live Vaccine Preparation, Immunization, and Mtb Challenge

Aliquots of the BCG-PEPf vaccine were stored in a freezer at −80 °C as previously described by Junqueira-Kipnis et al. 2013 [29]. The BCG-PEPf and BCG empty vector vaccines were thawed and adjusted to 1 × 10^7^ CFU/mL in 0.5% Tween 80 (PBST). Mice were subcutaneously vaccinated with BCG empty vector (*N* = 8) or BCG-PEPf (*N* = 8). Control groups were inoculated with PBST (*N* = 8). At 45 days after vaccination, the animals were challenged with 100 µL (10^6^ CFU) of *M. tuberculosis* by the intravenous route. To evaluate the recall of the immune response elicited by the vaccine, 30 days after the challenge, 4 animals from each group were euthanized, and the PEPf-specific lung T cells were evaluated. Forty-five days after the challenge, the bacterial load in the lungs of the remaining four mice from each group was determined. Also, at this time point, a lung lobe from each animal was used to evaluate the lung lesions. This experiment was repeated twice.

### 2.9. Booster Vaccine Strategy

To estimate the booster effect of the subunit vaccine after vaccination with BCG, 30 days after vaccination with BCG, the animals were intramuscularly boosted twice with the PEPf subunit vaccine composed of 20 mg of PEPf and 1 mg of Advax4 with 15-day intervals. This experiment was divided into four-mice groups: PBST, BCG, BCG+Advax4, and BCG boosted with PEPf + Advax4 subunit vaccines. Fifteen days after the last vaccination, the animals were challenged with *M. tuberculosis*, as described below. The protection induced by the vaccines was assessed 45 days after Mtb challenge. These experiments were repeated twice to assess reproducibility.

### 2.10. Mycobacterium Tuberculosis Infection

The virulent *M. tuberculosis* strain H37Rv was maintained as described by Junqueira-Kipnis et al. [29]. Mtb was briefly grown to a mid-log phase in a 7H9 medium containing OADC and 0.05% Tween 80, then frozen in aliquots in 20% glycerol at −80 °C for use as needed. One vial from the same batch was thawed and the inoculum adjusted to a concentration of 10^7^ CFU/mL by dilution in PBST for intravenous infection with 100 µL as described. One day after infection, one mouse from each group was euthanized, and the lung was collected, homogenized, and plated on 7H11 medium supplemented with OADC to determine the initial bacillary load in the lung. The infection resulted in a bacillary load of 10^5^ CFUs in the lungs of each animal at day 1.

### 2.11. Colony-Forming Unit (CFU) Determination of Infected Mice

The bacterial load of the infection was determined at 45 days post-infection as described above for different experiments. The lung homogenates were plated in OADC supplemented 7H11 medium. Animals were euthanized via cervical dislocation by a veterinarian, and the lungs were collected, homogenized, and plated in 7H11 medium supplemented with OADC. The bacterial load was determined by CFU count 21 days after incubation at 37 °C.

### 2.12. Histopathological Evaluations

The right caudal lung lobes of euthanized mice were collected after Mtb challenge according to each vaccination scheme’s times. The lungs were perfused with 0.05% heparin PBS by injection into the left ventricle of the heart. The right lower lobe was collected, conditioned in histological cassettes, and fixed with 10% buffered formalin. Samples were sectioned in 5 µm thick slices and stained with hematoxylin and eosin (HE) for microscopic analysis. Lesion scores were determined using AxionVision 4.9.1 software. Briefly, after acquiring 10 lung images with 10x magnification, all lesion areas identified in a single lung were summed, and the percentage of the lesion areas was calculated in relation to the total area of the visual field. Results are presented as percentages of injured areas.

### 2.13. Immune Response Analysis by Flow Cytometry

**Scheme 1:**
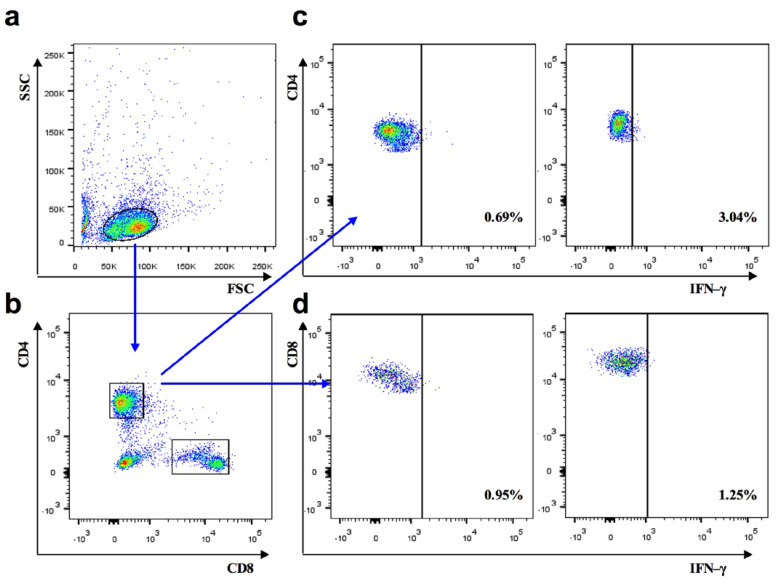
Gating strategy. (**a**) Lymphocytes were gated according to their granularity (SSC) and size (FSC) of singlet’s events. (**b**) CD4^+^ and CD8^+^ cells were gated and (**c**) the percentage of IFN-γ^+^ T cells stimulated with medium and (**d**) IFN-γ^+^ stimulated with PEPf cells were determined for each population.

In order to assess the specific immune response induced by PEPf, 15 days after the last immunization with the PEPf + Advax4 vaccine or 30 days after Mtb challenge, as described above. The animals were sacrificed by cervical dislocation, and inguinal lymphatic nodules or lungs were collected. The lungs were treated with DNAse IV solution (30 µg/mL; Sigma-Aldrich, Sao Paulo, Brazil) and collagenase III (0.7 mg/mL; Sigma-Aldrich) for 30 min at 37 °C. The single-cell suspension obtained after passage through a 70 µm cell filter (BDBiosciences) was treated with lysis buffer (0.15 M NH_4_Cl and 10 mM KHCO_3_). After washing with RPMI, the concentration was adjusted to 1 × 10^6^/mL, and 200 µL were seeded in 96-well plates (Cell Wells) previously incubated with 10 µL of anti-CD3 (eBioscience; 1 µg/mL). Cells were treated with ConA (1 µg/mL), PEPf (10 µg/mL), or medium and cultured for 12 h at 37 °C in a CO_2_ incubator. Then, monensin (eBioscience, 3 mM) was added, and the cells were incubated for an additional 5 h. Plates were centrifuged, and cells were incubated with a FITC-conjugated anti-CD4 antibody (BD PharMingen, Sao Paulo, Brazil) and PE-conjugated anti-CD8 antibody (BDPharMingen) for 30 min. Then, cells were washed, fixed, and permeabilized using BDCytofix/Cytopermkit. Cells were further incubated for 30 min with anti-IFN-γ antibody conjugated to APC (eBioscience, Sao Paulo, Brazil) and with anti-IL-17-A antibody conjugated to PErCP (eBioscience). Cell suspensions were acquired using a Biosciences BD FACSVerse^TM^ system and analyzed with FlowJo 9.0 software. A total of 50,000 total events or at least 30,000 lymphocytes were acquired per sample. Lymphocytes were gated according to their granularity (SSC) and size (FSC) of singlet’s events. CD4^+^ and CD8^+^ cells were gated and the percentage of IFN-γ+ cells was determined for each population (Scheme 1).

### 2.14. Statistical Analysis

The results were tabulated in Excel (version 14.3.4, 2011) and Prism 4 software (Graphpad Software 9.0, San Diego, USA). The comparison results were evaluated via a T-test followed by a Mann-Whitney post-test (two-tailed). Differences with *p*-values < 0.05 were considered statistically significant.

## 3. Results

### 3.1. Epitope Predictions

**Figure 1:**
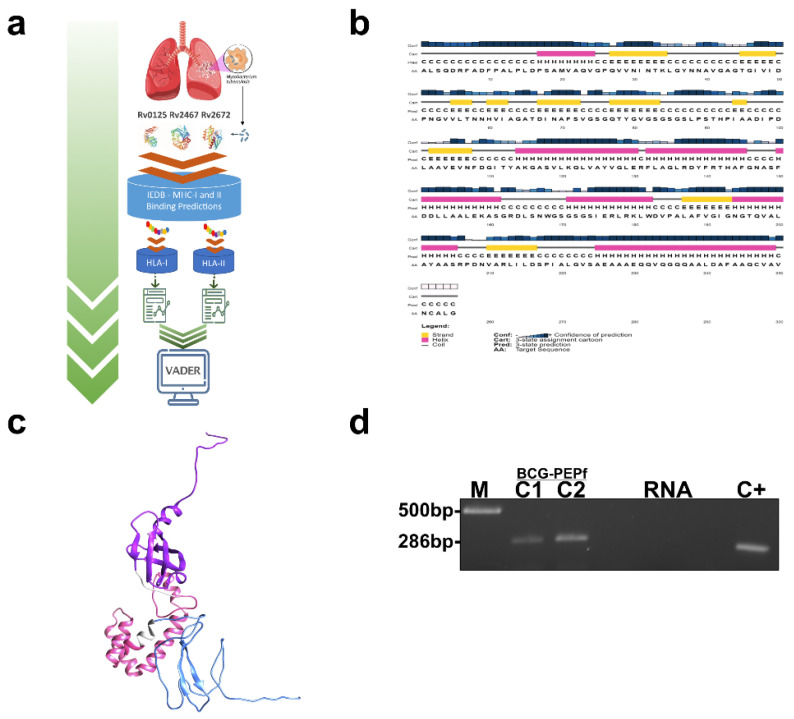
Construction of the PEPf fusion protein and its expression by live vaccines. (**a**) Prediction of HLA-I and -II binding epitopes and fusion protein design using the IEDB (Immune Epitope Database) and VADER (Vaccine-Associated Density Epitope Research) software. (**b**) PEPf is composed of 33% alpha-helices, 29% beta-sheets, and 37% loops. Yellow rectangles represent beta-sheets, pink rectangles represent the alpha-helices, and lines show the protein loops. (**c**) Tertiary structure of PEPf obtained using RaptorX and further refined using Galaxy Refine. (**d**) Expression of the recombinant fusion protein by the recombinant live vaccine, confirmed by extracting RNA from two recombinant BCG-PEPf clones (C1 and C2). The transcribed cDNA was amplified using specific primers for *pepf* flanking sequences. A plasmid carrying the fusion gene was used as a positive control (C+), and a cDNA synthesis reaction containing no reverse transcriptase (RNA) was used as a negative control. Lane M corresponds to 1 kb ladder from Sinapse Inc. (only the last band corresponding to 500 bp is shown).

After assessing the epitope density of each protease using VADER (Vaccine-Associated Density Epitope Research) software (Figure 1a), we selected the sequences with the highest epitope density. Figure 1b displays the amino acid sequence used to create the PEPf fusion protein. For PepA, the selected protein sequence ranging from amino acid residues 36–116 had 253 overlapping CD4 and 25 overlapping CD8 epitopes. Using the PepN protein, the selected sequence ranged from amino acid residues 360–440 and resulted in 491 overlapping CD4 and 44 overlapping CD8 epitopes. For Msh1, the sequence comprising amino acid residues 235–315 had 330 overlapping CD4 and 33 overlapping CD8 epitopes. The selected regions were spaced using the GSGSGS linker, originating a 26 kDa protein with 255 amino acids and with a theoretical pI of 4.7. The protein was predicted as stable via ProtParam, with an instability index of 19.17 (<40) and a grand average of hydropathicity (GRAVY) of 0.249. The secondary structure of PEPf had well-distributed percentages for alpha helices, beta sheets, and coils (loops) of 33%, 29%, and 37%, respectively (Figure 1c). The tertiary structure obtained using the RaptorX software showed an unnormalized global distance test (uGDT) > 50 (159) and GDT > 50 [30], indicating that the model was good.

### 3.2. PEPf Protein Used as a Subunit Vaccine Is Immunogenic in the Murine Vaccination Model

**Figure 2:**
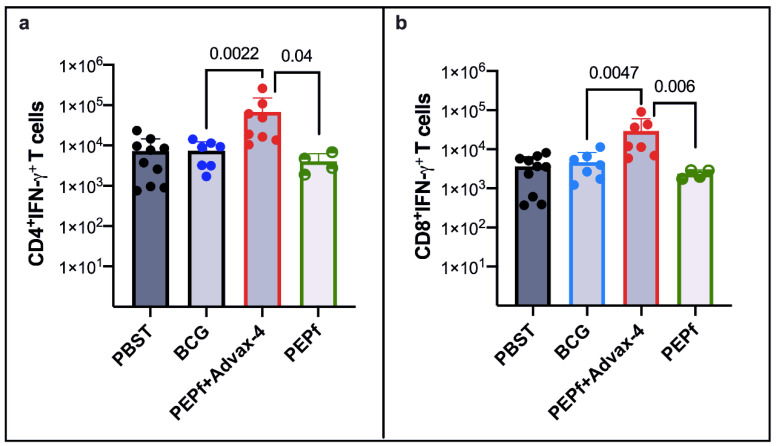
Immunogenicity of recombinant PEPf used with the Advax4 adjuvant. Fifteen days after the last immunization, lymph node cells from BALB/c PBST vaccinated (PBST, *N* = 8), BCG immunized (BCG, *N* = 8), with PEPf-Advax4 (PEPf + Advax4, *N* = 8), or PEPf alone (PEPf, protein control, *N* = 4) were analyzed. (**a**) CD4^+^IFN-γ^+^ T cells and (**b**) CD8^+^IFN-γ^+^ PEPf-specific T cells in the draining lymph nodes were determined and plotted on a logarithmic scale. The *p*-value is shown above the compared bars between significantly different groups. Data shown (circles) represent the cell numbers for each animal, and the horizontal bar shows the average (with SD) for each group of two independent experiments.

Recombinant purified PEPf protein was obtained after expression in *E. coli*, and its immunogenic capacity was assessed by using a previously published subunit vaccine scheme with Advax4 as an adjuvant [31]. Fifteen days after the last immunization, the presence of CD4^+^ and CD8^+^ T cells specific for the PEPf protein were evaluated in the draining lymph nodes. PEPf-Advax4 vaccination induced higher numbers of CD4^+^IFN-γ^+^- and CD8^+^IFN-γ^+^-specific T cell responses than the BCG or PEPf vaccines alone (Figure 2a,b, respectively). Mice vaccinated with PEPf or BCG did not present a PEPf-specific immune response, similar to the unvaccinated group. These results demonstrate that the created fusion protein in the studied model is immunogenic.

### 3.3. BCG-PEPf Vaccine Recalls Th1-Specific Immune Response after the M. tuberculosis Challenge and Diminishes the Lung Lesion Areas Caused by Infection

**Figure 3:**
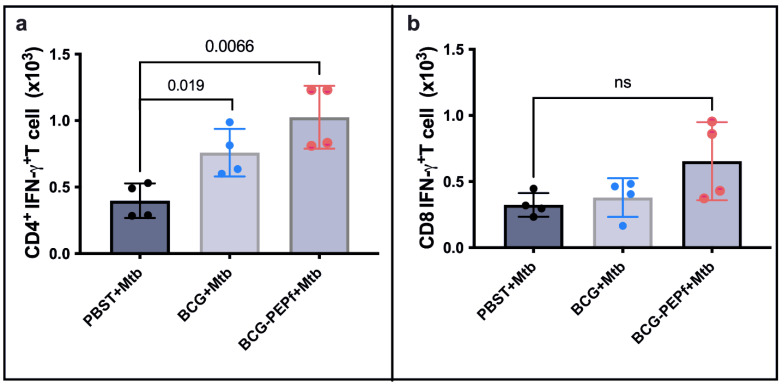
Immunogenicity of the BCG-PEPf vaccine. Forty-five days after immunization, mice were challenged using *M. tuberculosis*, and 30 days later, the lung cells from BALB/c PBST vaccinated (PBST + Mtb), immunized with BCG (BCG+Mtb), or BCG-PEPf (BCG-PEPf + Mtb) were analyzed after PEPf stimulation. (**a**) CD4^+^IFN-γ^+^ T cells and (**b**) CD8^+^IFN-γ^+^ PEPf-specific T cells in the lungs were determined. The *p*-value is shown above the compared bars between significantly different groups. Data shown (circles) represent the cell numbers for each individual animal, and the bars show the average (with SD) for each group. The results represent one of two independent experiments. ns, difference non-significant.

Since the fusion protein was shown to be immunogenic, it was utilized to test whether such a protein, when expressed by the BCG vaccine (Figure 1d), could induce the specific immune response requested in Mtb challenge. Mice were vaccinated with BCG or BCG-PEPf, and after 45 days, were challenged with Mtb [29,32]. Thirty days after the challenge, it was observed that specific CD4^+^IFN-γ^+^ T cell levels were higher in the lungs of animals vaccinated with BCG-PEPf compared to animals vaccinated with BCG (Figure 3a), indicating that the developed vaccine expressed the PEPf fusion protein in vivo, which was able to induce a specific immune response. None of the live vaccines were able to recall specific CD8^+^IFN-γ^+^ T cells upon Mtb infection (Figure 3b).

**Figure 4:**
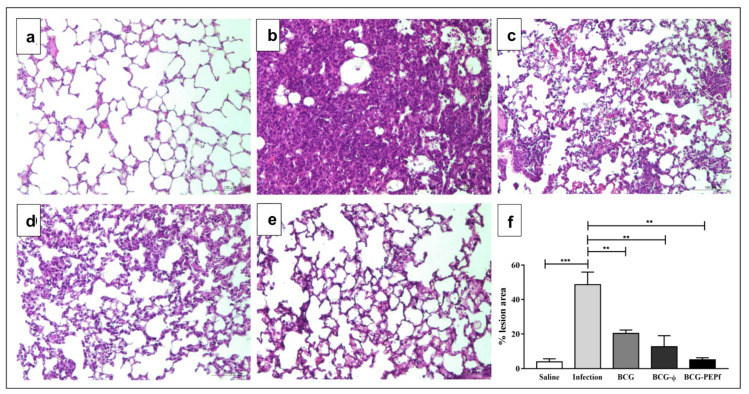
Immunization via the BCG-PEPf vaccine prevented the lung lesions caused by Mtb infection. Histopathological analysis of the lung slides stained with hematoxylin and eosin from the BALB/c mice 45 days after Mtb infection. (**a**–**e**) Representative slide sections at 200× magnification). (**a**) Control group without Mtb infection or vaccination (Saline); (**b**) Infected group (Infection); (**c**) Mice immunized with BCG and challenged with Mtb (BCG); (**d**) Mice immunized with BCG carrying an empty vector and challenged with Mtb (BCG-ϕ); (**e**) Mice immunized with BCG-PEPf and challenged with Mtb (BCG-PEPf); (**f**) Slides were analyzed via light microscopy, and the lung lesion scores (%) due to Mtb infection were determined. ** *p* < 0.01; *** *p* < 0.001 statistical significance between groups.

As expected, BCG vaccinations resulted in a significant reduction in lung lesion areas after Mtb challenge (Figure 4c–e) compared with the unvaccinated, infected control (Figure 4b). BCG-PEPf vaccinated animals had smaller areas of inflammatory infiltrates in the lungs (Figure 4e) when compared to animals vaccinated with parental BCG (Figure 4d), but the difference did not reach statistical significance.

**Figure 5:**
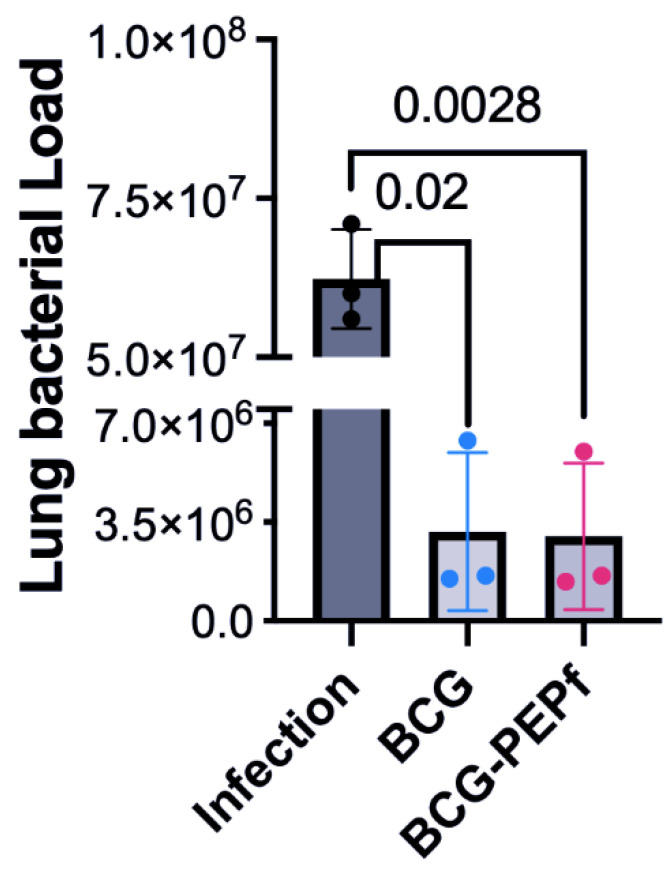
Protection against Mtb challenge after live vaccine immunizations. Forty-five days after challenging using Mtb, following immunization with BCG or BCG-PEPf, the mycobacterial loads in the lungs of the infected animals were determined (N = 4 mice per group). The bacterial loads in the vaccinated animal lungs were at least one log lower compared to unvaccinated animals. The protection conferred by BCG and recombinant BCG vaccines were similar. Data shown (circles) represent the CFUs for each animal, and the bar shows the average (with SD) for each group. The results represent one of two independent experiments.

Since BCG-PEPf showed a strong PEPf-specific CD4 T cell response after challenge with Mtb, we anticipated that the recombinant vaccine could also have improved Mtb growth restriction among infected mice compared to BCG. However, as shown in Figure 5, the BCG-PEPf vaccine failed to increase protection when compared to wt BCG.

### 3.4. Prime-Boost Vaccine Strategies Using BCG and PEPf + Advax4 Reduced Lung Lesions and Enhanced BCG Protection against Tuberculosis

**Figure 6:**
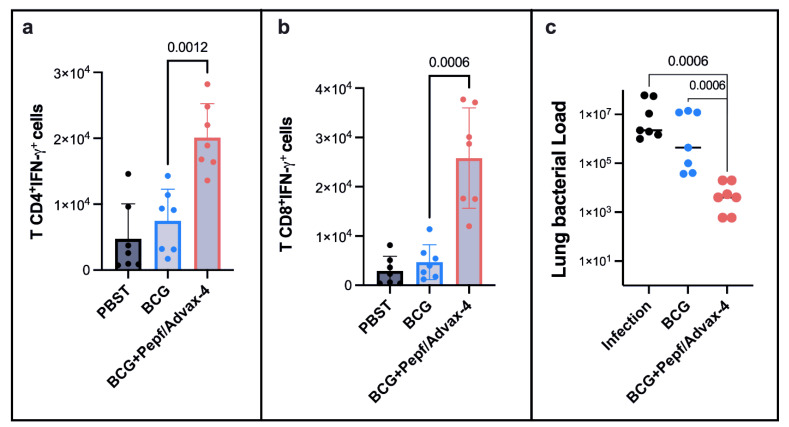
Prime-boost vaccine strategy using BCG and PEPf + Advax4-enhanced immune responses and protection against tuberculosis. Thirty days following immunization with BCG, mice were boosted using the PEPf-Advax4 subunit vaccine, and the specific immune response in the lungs was evaluated; (*N* = 7 mice, per group; (**a**,**b**)). Fifteen days after the last immunization, the mice were challenged with *M. tuberculosis* (**c**). The PEPf-Advax4 subunit vaccine induced higher numbers of CD4^+^IFN-γ^+^ (**a**) and CD8^+^IFN-γ^+^ (**b**)-specific T cells than both non-vaccinated and BCG-vaccinated mice. The bacterial loads in the animal lungs were determined for the non-vaccinated group (infected; *N* = 7), BCB-vaccinated (BCG; *N* = 7), and BCG-vaccinated, PEPf-Advax4-boosted (BCG+PEPf/Advax4; *N* = 7) subunit vaccine groups (**c**) and plotted on a logarithmic scale. The *p*-value is shown above the compared bars between significantly different groups. Data shown (circles) represent each animal, and the bar shows the average (with SD) for each group of two independent experiments.

Since the BCG vaccine did not induce PEPf-specific CD4^+^IFN-γ^+^ or CD8^+^IFN-γ^+^ T cells, it was reasoned that PEPf use as a BCG vaccine booster could improve BCG-induced protection against TB. Indeed, vaccination using the BCG plus PEPf-Advax4 vaccine strategy induced higher numbers of PEPf-specific CD4^+^IFN-γ^+^ and CD8^+^IFN-γ^+^ T cells (Figure 6a,b). Additionally, as shown in Figure 6c, the prime-boost strategy resulted in enhanced protection against Mtb challenge, although a similar reduction in lung lesions was observed among the vaccinated groups (data not shown).

## 4. Discussion

The main problem for TB control is related to the lack of an effective vaccine that triggers a protective immune response in adults. As such, it is essential to develop a new vaccine that can be an alternative to the BCG vaccine or used as a booster during adult life. Many studies have proposed that the absence of genes lost during *M. bovis* attenuation or the differential gene expression profile of the BCG vaccine could be responsible for the lack of the desired long-term protection, and thus, their reintroduction could enhance protection. Alternatively, some efforts are being made to improve vaccines by increasing the expression of key proteins (or epitopes) of Mtb/BCG [33]. *M. tuberculosis* expresses various proteases, some of which are critical for infection [e.g., Rv0125 (PepA), Rv2467 (PepN), and Rv2672 (Msh1)] and can be an immunogenic target for vaccine development.

In order to select the proteases to compose the developed fusion protein, the understanding that proteases exert specific functions that may contribute to the bacilli’s survival and virulence was taken into account; resultantly, proteases with sufficient genetic distances from the host to avoid autoimmune responses and with described immunological aspects were selected. Previous research demonstrated that PepA (Mtb32A), when used for PBMC stimulation of PPD+ patients, promotes the proliferation of mononuclear cells [11]. Additionally, Msh1 hydrolase is critical for Mtb survival in the host (foamy) macrophages’ hypoxic conditions; PepN-Mtb is also associated with several physiologically important bacillus proteins [14]. Furthermore, *Streptococcus pneumoniae* PepN proteins were shown to inhibit CD8^+^ T cell function [34].

This study presents a new fusion protein, PEPf, composed of high-density immunodominant epitope regions of the abovementioned three proteases. The selection considered whether the genes possessed conserved sequences in both Mtb and *M. bovis* (https://mycobrowser.epfl.ch/ (accessed on 14 February 2022)). PEPf was developed and used as a recombinant PEPf-expressing BCG or as a booster for BCG-induced immunity against tuberculosis. The prime-boost regimen following BCG vaccination conferred additional immunological advantages, which reduced lung lesions and enhanced protection against tuberculosis.

Several markers can be used to verify the efficacy of a vaccine, but the role of IFN-γ^+^-producing CD4^+^ T cells is already well-established in the protective immune response against *M. tuberculosis,* which is important for the control of bacterial growth in lung tissue [35,36], the restriction of Mtb growth in macrophages [37], the formation of granulomas [38,39], and the regulation of the Th17 profile [40]. Although the role of CD8^+^ T lymphocytes in TB is not as clear as that of CD4^+^ T lymphocytes, they are recognized as contributors to the generation of optimal and protective immunity [41,42]. Tc1 cells are capable of producing IL-2, IFN-γ, and TNF-α, cytokines recognized for playing critical roles during infection [4]. Therefore, the induction of Tc1 cells is a desirable feature in vaccines against tuberculosis. Recombinant PEPf combined with Advax4 was capable of inducing both CD4^+^ and CD8^+^ IFN-γ^+^-specific T cells (Tc1), suggesting its possible protective effect if used against Mtb infection.

The main difference observed between this strategy and BCG-PEPf vaccination was the induction of CD4^+^- and CD8^+^-specific T cells. The improvement in the production of CD4^+^ T cells expressing IFN-γ can be attributed to Advax4, which has CpG-DNA in its formulation. Notably, the addition of CpG-DNA to Advax4 increased the frequency of CD4+ T cells in comparison with Advax4 without the addition of CpG-DNA [43]. Furthermore, our group observed that a vaccine formulation containing Advax4 increased CD4^+^IFN-γ^+^ T cells significantly in comparison with a formulation without CpG-DNA [44]. The induction of the PEPf-specific CD8^+^ T cells shown in this study can be attributed to the protease sequences contained in PEPf combined with Advax4 (Figure 2). CD8^+^ T cells recognize short 8–10 amino acid peptides derived from cytosolic proteins presented by MHC class I [45]. The type of immune response generated by a protein subunit vaccine depends on the physical characteristics and location of the chosen antigen [46]. Although we do not know how these antigens are processed and presented, the observed response suggests that the selected high epitope density sequences favored both CD4^+^ and CD8^+^ T cell responses. In support of our findings, it was shown that the PepA (Mtb32a) strongly induces the production of IFN-γ^+^ by CD8^+^ T cells [47].

The pulmonary architecture of mice immunized with BCG-PEPf and challenged with Mtb was more preserved, with fewer lesions than BCG alone. Although other immune components important for Mtb control (e.g., Th17 and cytokines TNF-α and IL-2) were not analyzed, the immune response induced by BCG-PEPf displayed equivalent protection and better preservation of the affected organ. It is important to note that mice immunized with BCG alone and challenged with Mtb were able to induce CD4^+^ IFN-γ^+^ PEPf-specific T cells, whereas animals vaccinated with BCG or BCG-PEPf and challenged with Mtb did not induce a CD8^+^ T cell PEPf-specific response. As previously shown, BCG has a limited capacity to induce CD8^+^ T cell responses, which motivated the development of a new recombinant BCG vaccine, VPM1002 (currently in phase III clinical trial), to overcome this problem [48]. In contrast, the CD8^+^ T cell PEPf-specific response seen with PEPf + Advax4 could be due to a difference in antigen presentations or cytokine responses that avoided the induction of such cells induced by this strategy.

The PEPf vaccine, with the prime-boost strategy, significantly reduced the lungs’ bacillary loads compared to BCG vaccination alone. This bacillary reduction can be attributed to the induction of CD8^+^ IFN-γ^+^ PEPf-specific T cells that, in addition to producing cytokines, also release cytotoxic molecules—perforin, granzyme, and granulisin—helping to eliminate Mtb-infected cells [47].

This study has some limitations: for instance, the inability to reproduce identical human BCG and booster vaccination timelines in mice. However, such a strategy has been previously utilized in the prime-boost strategy [49]. Moreover, we previously showed [50] that BCG vaccination induced a CD4^+^ effector T cell response that persisted for 170 days. Here, we showed that BCG immunization did not induce a PEPf-specific response (Figure 2 and Figure 6).

## 5. Conclusions

The BCG-PEPf vaccine or the recombinant PEPf fusion protein used as a booster for the BCG vaccine conferred protection against Mtb challenge in mice. The protection was more expressive in the prime-boost strategy than the recombinant BCG vaccine. Such protection was likely achieved by the induction of CD8^+^IFN-γ^+^-specific T cells. The presented results hold promise for protease use in TB vaccine development.

## Data Availability

The data are available, upon request, from the corresponding author.

## Supplementary Materials

The following are available online at https://www.mdpi.com/article/10.3390/vaccines10020306/s1, Table S1: List of HLA I and HLA II used for the analysis of binding epitopes.
